# Novel mutations of *TCOF1 *gene in European patients with treacher Collins syndrome

**DOI:** 10.1186/1471-2350-12-125

**Published:** 2011-09-27

**Authors:** Chiara Conte, Maria Rosaria D'Apice, Fabrizio Rinaldi, Stefano Gambardella, Federica Sangiuolo, Giuseppe Novelli

**Affiliations:** 1 Fondazione Policlinico di Tor Vergata, Rome, Italy; 2 Dipartimento di Biopatologia e Diagnostica per Immagini, Università di Roma Tor Vergata, Rome, Italy; 3 Fondazione Livio Patrizi, Rome, Italy

**Keywords:** Treacher Collins syndrome, *TCOF1 *mutations, microdeletions, microinsertions

## Abstract

**Background:**

Treacher Collins syndrome (TCS) is one of the most severe autosomal dominant congenital disorders of craniofacial development and shows variable phenotypic expression. TCS is extremely rare, occurring with an incidence of 1 in 50.000 live births. The TCS distinguishing characteristics are represented by down slanting palpebral fissures, coloboma of the eyelid, micrognathia, microtia and other deformity of the ears, hypoplastic zygomatic arches, and macrostomia. Conductive hearing loss and cleft palate are often present. TCS results from mutations in the *TCOF1 *gene located on chromosome 5, which encodes a serine/alanine-rich nucleolar phospho-protein called Treacle. However, alterations in the *TCOF1 *gene have been implicated in only 81-93% of TCS cases.

**Methods:**

In this study, the entire coding regions of the *TCOF1 *gene, including newly described exons 6A and 16A, were sequenced in 46 unrelated subjects suspected of TCS clinical indication.

**Results:**

Fifteen mutations were reported, including twelve novel and three already described in 14 sporadic patients and in 3 familial cases. Moreover, seven novel polymorphisms were also described. Most of the mutations characterised were microdeletions spanning one or more nucleotides, in addition to an insertion of one nucleotide in exon 18 and a stop mutation. The deletions and the insertion described cause a premature termination of translation, resulting in a truncated protein.

**Conclusion:**

This study confirms that almost all the *TCOF1 *pathogenic mutations fall in the coding region and lead to an aberrant protein.

## Background

Treacher Collins syndrome (TCS; OMIM #154500) is an autosomal dominant disorder that affects the craniofacial development during early embryogenesis [1]. TCS is characterized by bilaterally symmetric features, including downward slanting palpebral fissures and colobomata of the lower eyelids, hypoplasia of the midfacial bones, cleft palate, and abnormal development of the external/middle ear that often leads to conductive hearing loss [2-4]. TCS occurs with an incidence of 1/50.000 and more than 60% of TCS cases has no previous family history and arises as the result of *de novo *mutations [5]. The syndrome is caused by mutations in the *TCOF1 *gene (OMIM #606847), which encodes the nucleolar phosphoprotein Treacle that may serve as a link between rDNA gene transcription and pre-rRNA processing [6]. Recently, Dauwerse et al. detected mutations in genes encoding subunits of RNA polymerases I and III (POLR1C and POLR1D) in Treacher Collins patients [7].

Thus far, most of the 200 disease-causing mutations described are deletions, insertions and nonsense, distributed along 28 exons [8]. Two additional exons have been reported: exon 6A, included in the most common isoform, and exon 16A, included in a minor isoform [9]. The mutations observed in TCS are predominantly sporadic, and the vast majority results in the introduction of a premature termination codon that can lead to the truncation of protein or to nonsense-mediated mRNA decay [10,11]. This suggests in the developmental anomalies result from haploinsufficiency of *TCOF1*. Penetrance of the genetic mutations underlying TCS is thought to be very high; however, extreme inter- and intra- familial phenotypic variation is reported [12].

In the present study, we screened 46 patients with a clinical diagnosis of TCS, by sequencing the entire *TCOF1* coding sequence together with the splice junctions. As result, 12 novel and 3 already reported mutations were characterised together with 7 novel and 13 known polymorphisms.

## Methods

### Patients

46 patients with a clinical diagnosis of TCS were recruited through several European Medical Institutes since 2002. In particular, the patients were evaluated at the University of Torino, Napoli, Rome Tor Vergata and "La Sapienza", University of Aquila, Ospedali Galliera of Genova, IRCCS Casa Sollievo della Sofferenza at San Giovanni Rotondo, Pediatric Department of SS. Pietro e Paolo Hospital at Borgosesia, Pediatric Department of the Bolzano Hospital, "Gaetano Rummo" Hospital at Benevento, Clinica Mangiagalli at Milano, S. Pietro Hospital at Rome, IRCCS "Ass. Oasi Maria SS" at Troina (Italy), GENDIA lab (Antwerp, Belgium), Egas Moniz Hospital (Lisbon, Portugal), Centre Hospitalier Universitaire de Rennes (France). Three patients have one parent with similar TCS features, while the other 43 cases haven't a family history. The major clinical features of TCS were recognized in all patients. After informed consent was obtained from patients or their families, blood samples were collected.

### Mutational analysis

Genomic DNA was obtained from peripheral blood samples using EZ1 DNA Blood 200 μl purification kit (Qiagen, GmbH, Germany). Coding regions and intron/exon boundaries of the *TCOF1 *gene were amplified in 28 reactions using specific primers [13]. For exons 6A, 10, 16A, 24, and 25 specific primers were self designed (Table 1).

**Table 1:** Primers self-designed for *TCOF1* gene amplification and sequencing

	FORWARD	REVERSE
Ex 6A	TTTATCAACTGCTGAAGCCCC	ATAGTCCTCCCTCTCCCCAAC

Ex 10	CTGAACCTAGAGCCCTGTGGG	AGACAGAGTCCCAGAGTGAGG

Ex 16A	TGGAAACCAGAGTGCCTGAG	TGATCCTGCAGCATCTGCAG

Ex 24	GCACCTCCCAACATTGAC	GAACCAGGTCTGGGTGT

Ex 25	TCACTAGTCCTCAGGAGGT	CTGCCTGGCTCTCTGGGA

PCR amplification was performed in a 25 μl reaction volume containing 2.5 U AmpliTaq Gold™ DNA polymerase (Applied Biosystems, Foster City, CA), 1X reaction buffer (10 mM Tris HCl pH 8.3, 50 mM KCl, 2.5 mM MgCl_2_), 200 μM of each deoxyribonucleoside triphosphate (dNTPs) and 0.2 mM each of primers using a PTC 100 thermocycler (MJ Research, Inc. Waltham, MA, USA).

A 10-minute denaturation step at 94°C was followed by 30 cycles at 94°C for 30 seconds, annealing temperature was performed, for each primer, 30 seconds at 52.5-62°C, and extending for 30 sec at 72°C; the reaction was completed by a final extension for 7 minutes at 72°C.

PCR products were purified by digestion with Antartic Phosphatase and Exonuclease I (New England BioLabs Inc.) and were sequenced in both directions using the Applied Biosystem Big Dye Terminator v3.1 Cycle sequencing kit.

New mutations were not found in 100 normal chromosomes by sequencing.

### Nucleotide and Aminoacid numbering

All mutations were named considering the genomic reference [NT_029289] and the cDNA that corresponds to the major treacle isoform [NM_001135243.1] [14]. Mutation nomenclature is based on HGVS nomenclature guidelines [http://www.hgvs.org/mutnomen] [15].

### *In silico *tools

The splice predictor software program, NNSplice version 0.9 [http://www.fruitfly.org/seq_tools/splice.html], was used for an initial approach to novel variant (c.IVS16A-30G→A) suspected of causing aberrant RNA processing of the *TCOF1 *gene. The ESE finder 2.0 program [http://rulai.cshl.edu/tools/ESE2/] was used to predict hypothetical splicing enhancer in mutated c.IVS16A-30G→A sequence.

## Results

**Figure 1:**
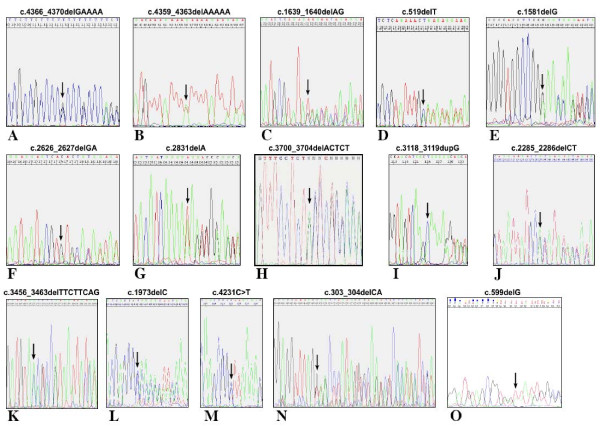
**A-O Chromatograms of characterized pathogenic mutations in TCS patients**. The header of each picture indicates the nucleotide mutation. The arrows show the site of mutation in chromatograms. A and E chromatograms report reverse sequences; B-D and F-O chromatograms report forward sequences.

The *TCOF1 *gene analysis was carried out on the 46 TCS European patients by direct sequencing of 28 PCR genomic fragments encompassing the complete coding sequence and splice sites. We detected 15 different *TCOF1 *mutations in 17 patients (Table 2 Figure 1), 12 new and 3 already described.

**Table 2:** Pathogenic mutations in TCS patients

PATIENT	GEOGRAPHIC ORIGIN	EXON	cDNA MAJOR ISOFORM MUTATION	PROTEIN MUTATION	OCCURRENCE	REFERENCE
TCS 1	caucasian	3	c.303_304delCA	p.A101Afsx73	Sporadic	In this study

TCS 2	caucasian	5	c.519delT	p.T173Tfsx46	Sporadic	In this study

TCS 3	caucasian	6	c.599delG	p.S200Tfsx19	Familial	In this study

TCS 4	caucasian	10	c.1639_1640delAG	p.S547Qfsx2	Sporadic	[13]

TCS 5	caucasian	10	c.1581delG	p.G587Gfsx69	Sporadic	In this study

TCS 6	caucasian	12	c.1973delC	p.P658Lfsx53	Sporadic	In this study

TCS 7	caucasian	13	c.2285_2286delCT	p.S762fs	Sporadic	In this study

TCS 8	caucasian	15	c.2626_2627delGA	p.D876Qfsx2	Sporadic	In this study

TCS 9	caucasian	16	c.2831delA	p.E944Efsx6	Sporadic	In this study

TCS 10	caucasian	18	c.3118_3119dupG	p.A1040Gfx47	Familial	In this study

TCS 11	caucasian	20	c.3456_63delTTCTTCAG	p.S1152Rfsx3	Sporadic	In this study

TCS 12	caucasian	22	c.3700_3704delACTCT	p.T1234Gfsx5	Sporadic	In this study

TCS 13	caucasian	23B	c.4331C > T	p.Q1411X	Sporadic	In this study

TCS 14	caucasian	24	c.4359_4363delAAAAA	p.E1453Efsx16	Sporadic	[17]

TCS 15 TCS 16 TCS 17	caucasian	24	c.4366_4370delGAAAA	p.E1456Efsx13	Familial Sporadic Sporadic	[17]

The cDNA major isoform (including exon 6A, but without exon 16A) was deposited in GenBank with accession number AY460334[14].

For the cDNA sequence, nucleotide +1 is the A of the ATG-translation initiation codon.

Considering the already known mutations, the 5-bp deletion, c.4366_4370delGAAAA, in exon 24 (Figure 1A), was the most frequent mutation observed in this study (3/17, 17%) (Patient TCS 15,16 and 17). In the same exon (patient TCS 14), we identified the already known frameshift mutation c.4359_4363delAAAAA (Figure 1B). Both the mutations identified fall within the *TCOF1 *mutation hot spot rich in 18 Lysine residues. Other microdeletions have already been described in this region [8,11,16-18].

The other known mutation is a 2 bp deletion, c.1639_1640delAG (patient TCS 4), localized in exon 10 (Figure 1C) [8,13].

A total of 12 novel disease-causative mutations were found in 2 familial cases and in 10 sporadic TCS patients. These consist of 10 microdeletions (sized 1/15 nucleotides), one single-base duplication, and one nonsense mutation. All deletions and insertion cause a frameshift and produce a truncated protein.

The first reported pathogenic microdeletion was a c.519delT in exon 5 (patient TCS 2) (Figure 1D) leading to formation of a stop codon 46 aminoacids later. This alteration was not found in the patient's parents. Patient TCS 5 was found to bear a *de novo *truncating mutation, c.1581delG, in exon 10 (Figure 1E) that introduces a premature stop codon 69 aminoacids later.

Two alterations were identified in exons 15 and 16, that encode repetitive motifs. The former, c.2626_2627delGA in exon 15 (Figure 1F), was identified in patient TCS 8 and causes the formation of a stop codon two aminoacids later. The latter, c.2831delA (Figure 1G), identified in the exon 16 of patient TCS 9, causes a frameshift.

Another proband (TCS 12) was heterozygous for the *de novo *c.3700_3704delACTCT mutation in exon 22 (Figure 1H).

The sequence analysis of a DNA sample obtained from patient TCS 10 identified the c.3118_3119dupG mutation in exon 18 (Figure 1I). Analysis of the relatives indicated that this duplication was also present in the affected father and brother.

Two more *TCOF1 *variations were found in patient TCS 7. The first one was a single base sostitution, c.2924C > T, in exon 17, determining the aminoacidic change p.P975L. The other one was a 2 bp deletion, c.2285_2286delCT in exon 13 (Figure 1J), wich results in a frameshift and premature stop codon. Neither abnormality was found in 140 and 100 healty chromosomes, respectively, indicating that they were not common polymorphisms.

Analysis of TCS 7's normal parents DNA showed the mother was a carrier of c.2924C > T. Neither of parents showed the 2 bp deletion. Thus, it was indicated that the 2 bp deletion, disrupting protein translation, is probably the causative mutation.

In the TCS 11 patient, a truncating mutation of 8 bases c.3456_3463delTTCTTCAG (Figure 1K) was found in exon 20, causing a frameshift resulting in a stop codon four codons later. The same kind of mutation was found in TCS 6 patient in exon 12. A microdeletion of one single base c.1973delC (Figure 1L) with a stop codon 52 codons later.

In patient TCS 13 a nonsense mutation c.4231C > T (Figure 1M) was found. This pathogenetic variation occurred in exon 23B causing a stop codon (p.Q1411X). In patient TCS 1 a small deletion was found. It was a truncating mutation of the last 2 nucleotides of exon 3, c.303_304delCA (Figure 1N), causing a stop codon 73 codons later. Actually, the deletion involves the first 13 nucleotides of intron 3 (IVS3+13delGTAAGAGCCTTGC), too.

Finally, we detected a one bp deletion c.599delG in patient TCS 3 (Figure 1O). It was a familial case, as we confirmed the mutation in proband's son and daughter. It was located in exon 6 and it caused a stop codon 19 codons later.

In 29 patients, with apparently TCS phenotype, no pathogenic mutations have been identified after screening of the whole coding region of the gene. However these patients present clinical features of the syndrome. All these cases are isolated.

A large number of *TCOF1 *polymorphisms were detected. Thirteen of these were already published and 7 are novel (Table 3). Twelve were silent or missense variation and 8 occur in intronic regions. All novel polymorphisms were present in controls in different percentages.

**Table 3:** Polymorphisms in TCS patients

PATIENT	LOCATION	cDNA MAJOR ISOFORM POLYMORPHISM	AMINOACID CHANGE	ALLELE FREQUENCIES IN CONTROLS	REFERENCE
TCS 8, TCS 18	5'UTR	5'UTR-41G > T	None	0.82	In this study
TCS 18	Intron 6	c..639 +32C > G	None	0.02	[11]
TCS 19	Exon 7	c.1347T > C	p.P439L	0.01	[13]
TCS 6, TCS 11	Exon 10	c.1578 T > C	p.P526P	0.77	[13]
TCS 1, TCS 6, TCS 17, TCS 19	Exon 11	c.1761G > T	p.G587G	0.10	[11]
TCS 17, TCS 19, TCS 20	Exon 11	c.1842A > G	p.S614S	0.52	[13]
TCS 10	Exon 12	1837G > C	p.A588P	0.31	In this study
TCS 10, TCS 10.1, TCS 10.3, TCS 11, TCS 20	Exon 12	c.1993C > G	p.A665P	0.21	International HapMap project
TCS10, TCS 11, TCS 20	Intron 15	c.2659-28delTCTC	None	0.15	In this study
TCS 13	Exon16	c.2660 C > T	p.A887V	0.82	[13]
TCS 19	Exon 16	c.2765 C > T	p.S992L	0.01	[13]
TCS 21	Intron 16	c.2859-30G > A	None	0.0	In this study
TCS 11, TCS 20	Intron 16A	c.2859+3444C > T	None	0.05	In this study
TCS 7, Mother carrier	Exon 17	c.2924C > T	p.P975L	0.0	In this study
TCS 22	Intron 19	c.3197+66C > T	None	1.0	International HapMap project
TCS 13, TCS 19	Intron 20	c.3517-34G > A	None	0.1	In this study
TCS 13, TCS 14, TCS19, TCS 23, TCS 24, TCS 25	Exon 21	c.3527C > G	p.P1176R	0.16	[11]
TCS 1, TCS 13, TCS 19	Intron 21	c.3370-3C > T	None	1.0	[8]
TCS 6, TCS 22, TCS 26, TCS 27, TCS 28,TCS 29, TCS 30, TCS 31, TCS 32	Exon 23B	c.4169C > T	p.A1390V	0.24	[11]
TCS 23, TCS 32	Exon 23B	C.4292G > C	p.G1431A	0.03	[11]

The URL for International HapMap project is http://hapmap.ncbi.nlm.nih.gov/

Patient TCS 21 had a single base substitution c.2859-30G > A in exon 16. This substitution wasn't identified in 150 healthy chromosomes; therefore, this variation could be a novel splicing mutation in the *TCOF1 *gene. Computer simulation studies were performed to evaluate the role of this variation in hypothetical splicing enhancer or splice site prediction, and we deduced that the mutated sequence determines a loss of SF2/ASF and Srp55 and a gain of SRp40 binding sites, but the same splice site strength score was 0.89, similar to wild type sequence, according to ESE finder and NNSplice programs, respectively.

## Discussion

In this study, we report the screening of entire *TCOF1 *coding region and the identification of a spectrum of 3 known mutations, 12 novel pathogenetic mutations and 7 novel polymorphisms by direct sequencing. In all familial cases, we identified the *TCOF1 *mutation in one parent with similar TCS features. Of the 43 analyzed sporadic cases, 14 had arisen as the result of a *de novo *mutations in the *TCOF1 *gene. The sensitivity of sequencing analysis of *TCOF1 *gene on our patients was 37% (17/46). The remaining 29 TCS patients, negative to the *TCOF1 *screening, have to be clinically revaluated. In this regard, we recommend a clinical revaluation using craniofacial radiographs, extremely useful in detecting zygomatic hypoplasia as a clinical feature of TCS patients [19]. On the other hand, the differential diagnosis is necessary. In fact, Nager and Miller syndrome exhibits phenotypic overlap with TCS. Moreover, mutations in genes encoding subunits of RNA polymerases I and III (POLR1C and POLR1D) cause TCS, too [7]. We are considering to perform the screening of POLR1C and POLR1D genes in our negatives *TCOF1* patients.

The *TCOF1 *gene mutations include missense, nonsense, small deletions and duplications. In particular, the most common classes of *TCOF1 *alleles are small deletions (60%) and duplications (25%) resulting in frameshifts [10]. According to literature data, 76.5% (13/17) of our characterised mutations are microdeletions and 6% (1/17) are duplications. Though it has been suggested that five exons (10, 15, 16, 23 and 24), are defined as a hot spot region of the *TCOF1 *gene mutations [14], a distribution of pathogenic variations along all the gene was reported by different authors. We confirmed exon 24 as hot spot of the *TCOF1 *gene as we described the major number of mutations in the exon (Table 2 Figure 1). The c.4366_4370delGAAAA is the most frequent mutation as we found it in three of 16 affected patients. This is probably due to the high repetition of adenines (60% of exon) making exon 24 region prones to polymerase slippage in DNA replication [14]. Moreover, the high complexity of this exon make it difficult to give the right nomenclature to the identified sequence variations. It is therefore mandatory to sequence exon 24 in both directions. Also in this study exons 10, 15, and 16 were revealed as pathogenic gene regions. Finally, three mutations were found in rarely affected sequences.

Two more *TCOF1 *variations were found in patient TCAR. The presence of two possible *TCOF1 *mutations in the same patient has been reported in a paper by Fujioka H et al. [20]. In this case, familiar analysis is requested to predict which is the benign and which is the pathological variation in TCS.

All patients, and in particular patients with typical TCS features but negative *TCOF1 *screening, were analyzed for the two alternatively spliced in-frame exons (6A and 16A) and no mutations were found.

## Conclusion

In this work, the observation of affected features, combined with a molecular analysis, is sufficient to perform a correct TCS diagnosis in 35% of cases. This is the phenotypic variability of TCS.

## Competing interests

The authors declare that they have no competing interests.

## Authors' contributions

CC, MRD decided the study; CC, FR collected blood samples and performed the experiments; SG, FS wrote the paper, GN coordinated this study. All authors read and approved the final manuscript.

## Pre-publication history

The pre-publication history for this paper can be accessed here:

http://www.biomedcentral.com/1471-2350/12/125/prepub

## Ackowledgements

We are thankful to Drs. Silengo M, David V, Blayau M, Guala A, Dalla Monica M, Scarano G, Bengala M, Frontali M, Willems P, Novelli A, Sorrentino, Calcagno G, Lourenço MT, Dagna Bricarelli F, Faravelli F, Tozzi MC, Ligas C, Zelante L, Castellan C, Stanzial F, Lalatta F, and Avola E for referring their patients for evaluation to us. We also thank Gigante L, Longo G, Nuovo S for help with mutation screening.

## References

[B1] Gorlin RJ Cohen MM Levin LS Syndromes of the head and neck Oxford University Press, 1990, Oxford, UK

[B2] Treacher Collins E Case with symmetrical congenital notches in the outer part of each lower lid and defective development of the malar bones Trans Opthalmol Soc UK 1900 20 90

[B3] Franceschetti A Klein D The mandibulofacial dysostosis; a new hereditary syndrome Acta Ophthalmol 1949 27 143 224 18142195

[B4] Phelps PD Poswillo D Lloyd GA The ear deformities in mandibulofacial dysostosis (Treacher Collins syndrome) Clin Otolaryngol Allied Sci 1981 6 15 28 10.1111/j.1365-2273.1981.tb01782.x 7273449

[B5] Splendore A Jabs EW Felix TM Passos-Bueno MR Parental origin of mutations in sporadic cases of Treacher Collins syndrome Eur J Hum Genet 2003 11 718 722 10.1038/sj.ejhg.5201029 12939661

[B6] The Treacher Collins Syndrome Collaborative Group Positional cloning of a gene involved in the pathogenesis of Treacher Collins syndrome Nat Genet 1996 12 130 136 8563749 10.1038/ng0296-130

[B7] Dauwerse JG Dixon J Seland S Ruivenkamp CA van Haeringen A Hoefsloot LH Peters DJ Boers AC Daumer-Haas C Maiwald R Zweier C Kerr B Cobo AM Toral JF Hoogeboom AJ Lohmann DR Hehr U Dixon MJ Breuning MH Wieczorek D Mutations in genes encoding subunits of RNA polymerases I and III cause Treacher Collins syndrome Nat Genet 2011 43 20 22 10.1038/ng.724 21131976

[B8] Splendore A Jabs EW Passos-Bueno MR Screening of TCOF1 in patients from different populations: confirmation of mutational hot spots and identification of a novel missense mutation that suggests an important functional domain in the protein treacle J Med Genet 2002 39 493 495 10.1136/jmg.39.7.493 12114482 PMC1735178

[B9] So BR Gonzales B Henning D Dixon J Dixon MJ Valdez BC Another face of the Treacher Collins syndrome (TCOF1) gene: identification of additional exons Gene 2004 328 49 57 15019983 10.1016/j.gene.2003.11.027

[B10] Dixon MJ Marres HA Edwards SJ Dixon J Cremers CW Treacher Collins syndrome: correlation between clinical and genetic linkage studies Clin Dysmorphol Apr 1994 3 96 103 8055143

[B11] Splendore A Silva EO Alonso LG Richieri-Costa A Alonso N Rosa A Carakushanky G Cavalcanti DP Brunoni D Passos-Bueno MR High mutation detection rate in TCOF1 among Treacher Collins syndrome patients reveals clustering of mutations and 16 novel pathogenic changes Hum Mutat 2000 16 315 322 10.1002/1098-1004(200010)16:4<315::AID-HUMU4>3.0.CO;2-H 11013442

[B12] Dixon J Ellis I Bottani A Temple K Dixon MJ Identification of mutations in TCOF1: use of molecular analysis in the pre- and postnatal diagnosis of Treacher Collins syndrome Am J Med Genet A 2004 127A 244 248 10.1002/ajmg.a.30010 15150774

[B13] Wise CA Chiang LC Paznekas WA Sharma M Musy MM Ashley JA Lovett M Jabs EW TCOF1 gene encodes a putative nucleolar phosphoprotein that exhibits mutations in Treacher Collins Syndrome throughout its coding region Proc Natl Acad Sci USA 1997 94 3110 3115 10.1073/pnas.94.7.3110 9096354 PMC20330

[B14] Splendore A Fanganiello RD Masotti C Morganti LS Passos-Bueno MR TCOF1 mutation database: novel mutation in the alternatively spliced exon 6A and update in mutation nomenclature Hum Mutat 2005 25 429 434 10.1002/humu.20159 15832313

[B15] den Dunnen JT Antonarakis SE Mutation nomenclature extensions and suggestions to describe complex mutations: a discussion Hum Mutat 2000 15 7 12 10.1002/(SICI)1098-1004(200001)15:1<7::AID-HUMU4>3.0.CO;2-N 10612815

[B16] Teber OA Gillessen-Kaesbach G Fischer S Böhringer S Albrecht B Albert A Arslan-Kirchner M Haan E Hagedorn-Greiwe M Hammans C Henn W Hinkel GK König R Kunstmann E Kunze J Neumann LM Prott EC Rauch A Rott HD Seidel H Spranger S Sprengel M Zoll B Lohmann DR Wieczorek D Genotyping in 46 patients with tentative diagnosis of Treacher Collins syndrome revealed unexpected phenotypic variation Eur J Hum Genet 2004 12 879 890 10.1038/sj.ejhg.5201260 15340364

[B17] Edwards SJ Gladwin AJ Dixon MJ The mutational spectrum in Treacher Collins syndrome reveals a predominance of mutations that create a premature-termination codon Am J Hum Genet 1997 60 515 524 9042910 PMC1712503

[B18] Ellis PE Dawson M Dixon MJ Mutation testing in Treacher Collins Syndrome J Orthod 2002 29 293 297 10.1093/ortho/29.4.293 12444270

[B19] Marres HA Cremers CW Dixon MJ Huygen PL Joosten FB The Treacher Collins syndrome: correlation between clinical and genetic linkage study on two pedigrees Arch Otolaryngol Head Neck Surg 1995 121 509 514 7727083 10.1001/archotol.1995.01890050009002

[B20] Fujioka H Ariga T Horiuchi K Ishikiriyama S Oyama K Otsu M Kawashima K Yamamoto Y Sugihara T Sakiyama Y Detection of a novel silent deletion, a missense mutation and a nonsense mutation in TCOF1 Pediatr Int 2008 50 806 809 10.1111/j.1442-200X.2008.02650.x 19067896

